# A Role for Selection in the Evolution of Genetic Robustness

**DOI:** 10.1371/journal.pbio.0030407

**Published:** 2005-11-01

When Darwin proposed the interplay between variation and natural selection as the driving force of evolution, he had no idea what material produced that variation. Ninety-one years later, Hershey and Chase's famous blender experiment identified the source of variation as DNA. Today, biologists are still struggling to elucidate the details of that interplay.

Natural selection works on genetic variations that produce physical changes in an organism. Think of an organism as a collection of genes (its genotype), and its physical characteristics (its phenotype) as its genotype interfacing with the environment, a natural laboratory that saves or discards a genotype based on the performance of its phenotype. Assuming that a population of genotypes is well adapted to its environment, most mutations are likely to reduce performance in that environment. Thus, populations at equilibrium should experience selection for mechanisms that protect the phenotype against mounting deleterious mutations—a phenomenon called mutational (or genetic) robustness. Most of the evidence that robustness arises from selection rather than chance comes from theoretical studies and from studies of “digital organisms”—computer programs that self-replicate, mutate, and evolve—and has proved difficult to establish in the lab. [Fig pbio-0030407-g001]

**Figure pbio-0030407-g001:**
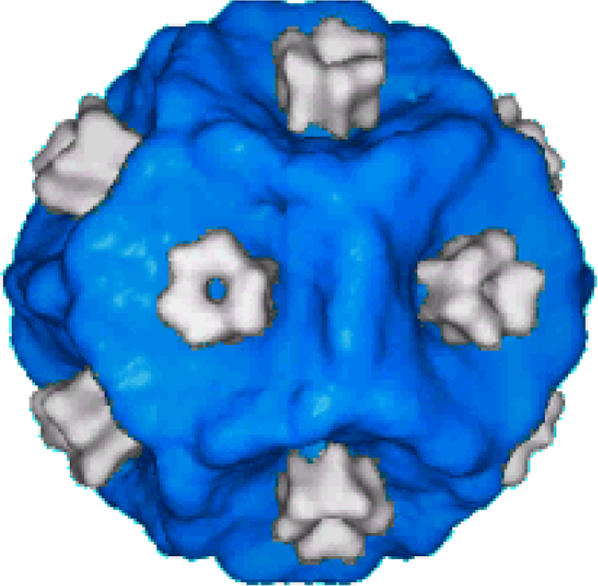
RNA phage viruses that co-infect host cells with more fit viruses can withstand high mutation loads with the help of their fitter counterparts—but only for so long

In a new study, Rebecca Montville, Paul Turner, and their colleagues provide experimental evidence for adaptive genetic robustness by working with a mutation-prone virus that infects bacteria, called RNA phage ψ6. (Viruses that infect bacteria are called phages.) Though theoretical predictions for mutational robustness assume that phenotype expression results solely from the underlying genotype, many viruses can overcome their own mutational deficiencies by co-opting the proteins produced by more fit viruses co-infecting the same host, a feature called complementation.

In a previous study, the authors demonstrated for RNA phage ψ6 that complementation buffers less fit viruses against the harmful effects of mutations. They then created six replicate populations of phages and allowed them to adapt to their bacterial host over hundreds of virus generations; three populations evolved at a low ratio of infecting viruses to bacteria (called low multiplicity of infection [MOI]) and three at a high MOI. Populations at high MOI experienced higher rates of co-infection. In this study, the authors investigated the evolutionary consequences of this phenomenon with the hypothesis that selection for mutational robustness should be relaxed for co-infecting phages, since phenotype constancy is bolstered by co-infection with their fitter viral companions.

Using clones from their six replicate populations, Montville et al. generated 60 new lineages and subjected them to a mutation accumulation experiment under conditions that allowed mutations to accumulate at roughly the same probability in high and low MOI lineages. The authors then evaluated the fitness consequences of mutation accumulation on the lineages by comparing their growth rate in the bacteria before and after mutation accumulation. The authors found greater variance in fitness change for the high co-infection lineages compared to the low-infection lineages, supporting the hypothesis that selection for mutational robustness is stronger in the absence of co-infection.

The authors go on to rule out the notion that different fitness effects were produced by different mutation rates in the lineages. Interestingly, the less robust viral genomes were copied more accurately than their more robust counterparts; why less accurate genome replication might accompany the evolution of robustness is a question for future study.

While complementation appears to buffer the damage of mutational onslaughts in the short-term, this benefit of co-infection eventually disappears because the buffer slows the rate that harmful mutations are culled from the virus population. The authors highlight an additional cost of co-infection: by weakening selection for robustness, co-infection may favor the evolution of genomes that are more vulnerable to the harmful effects of mutation. Future work is needed to examine this seeming tug-of-war between short-term and long-term consequences of co-infection, and its impact on the evolution of virus traits. —*Liza Gross*

